# Crystallographic and SAXS studies of *S*-adenosyl-l-homocysteine hydrolase from *Bradyrhizobium elkanii*

**DOI:** 10.1107/S2052252517002433

**Published:** 2017-04-10

**Authors:** Tomasz Manszewski, Kamil Szpotkowski, Mariusz Jaskolski

**Affiliations:** a Center for Biocrystallographic Research, Institute of Bioorganic Chemistry, Polish Academy of Sciences, Poznan, Poland; b Department of Crystallography, Faculty of Chemistry, A. Mickiewicz University, Poznan, Poland

**Keywords:** SAH, SAM, SAHase adenosine, adenine, 2′-deoxyadenosine, 3′-deoxyadenosine, cordycepin, homocysteine, nicotinamide adenine dinucleotide, NAD, molecular gate

## Abstract

*S*-Adenosyl-l-homocysteine hydrolase (SAHase) from the symbiotic bacterium *Bradyrhizobium elkanii* (BeSAHase) was crystallized in four ligand complexes with (i) mixed adenosine (Ado) and cordycepin (Cord; 3′-deoxyadenosine), (ii) adenine (Ade), (iii) Ado and (iv) mixed 2′-deoxyadenosine (2′-dAdo) and Ade. The crystal structures were solved at resolutions of 1.84, 1.95, 1.95 and 1.54 Å, respectively. Only the Ade complex crystallized with a dimer in the asymmetric unit, while all of the other complexes formed a crystallographically independent tetrameric assembly. In the Ado/Cord complex, adenosine is found in three subunits while the fourth subunit has cordycepin bound in the active site. In the Ade and Ado complexes only these ligand molecules are present in the active sites. The 2′-dAdo/Ade complex has Ade bound in two subunits and 2′-dAdo bound in the other two subunits. The BeSAHase fold adopted a closed conformation in the complexes with Ado, Ade and 2′-dAdo, and a semi-open conformation when cordycepin occupied the active site. An SAHase-specific molecular gate, consisting of residues His342 and Phe343, behaves differently in the different complexes, but there is no simple correlation with the ligand type. Additional small-angle X-ray scattering (SAXS) experiments confirm the tetrameric state of the protein in solution. The main conclusions from this work are (i) that the SAHase subunit does not simply oscillate between two discrete conformational open/closed states in correlation with the absence/presence of a ligand in the active site, but can also assume an intermediate form for some ligands; (ii) that the shut/open state of the molecular gate in the access channel to the active site is not correlated in a simple way with the open/closed subunit conformation or empty/occupied status of the active site, but that a variety of states are possible even for the same ligand; (iii) that a cation (typically sodium) coordinated in an intersubunit loop rigidifies a molecular hinge and thus stabilizes the closed conformation; (iv) that BeSAHase in solution is a tetramer, consistent with the model derived from crystallography.

## Abstract 2

Tetrameric *S*-adenosyl-l-homocysteine hydrolase from a nitrogen-fixing bacterium was crystallized in complexes with four different ligands. The crystal structures reveal that there is no simple correlation between the cargo molecule in the active site and the open/closed subunit conformation or the open/shut state of the active-site access gate.

## 1. Introduction   

*S*-Adenosyl-l-methionine (SAM)-dependent methyltrans­ferases are inhibited by *S*-adenosyl-l-homocysteine (SAH), a byproduct of the methylation reaction (Chiang & Cantoni, 1979 ▸; Cantoni & Chiang, 1980 ▸; Liu *et al.*, 1992 ▸). SAM is the most commonly used substrate for cellular methylation processes of a variety of compounds (Cantoni, 1975 ▸; Richards *et al.*, 1978 ▸). SAH, which is formed upon methyl-group transfer from SAM, must be removed from the reaction equilibrium (which is shifted far towards SAH formation) by its hydrolase (SAHase; De La Haba & Cantoni, 1959 ▸), which splits it into Ado and homocysteine (Hcy). In this way, SAHase is a key regulator of SAM-dependent methylation reactions and of Hcy levels, which, when elevated, are a risk factor for neurodegenerative diseases, neural tube defects and cardiovascular diseases (Jakubowski, 2006 ▸).

The mechanism of SAH hydrolysis catalyzed by SAHase was first elucidated by Palmer & Abeles (1976 ▸, 1979 ▸). It assumes the formation of a 3′-keto derivative *via* oxidation of SAH by NAD^+^, followed by H3′ proton abstraction from the ribose moiety by the N^ζ^ atom of a catalytic lysine residue and by Hcy elimination. After a Michael-type addition of a water molecule, the final product is reduced by NADH, which is regenerated to NAD^+^.

SAHases are present in all kingdoms of life, although their sequences show notable differences, usually in the form of kingdom-specific insertions or deletions (Stępkowski *et al.*, 2005 ▸). Most SAHases function as homotetramers, although the enzymes from plants have been shown to be active as dimers (Guranowski & Pawełkiewicz, 1977 ▸; Brzezinski *et al.*, 2008 ▸). For enzymatic activity, the protein requires one NAD^+^ cofactor molecule bound in each subunit near the active site (Palmer & Abeles, 1979 ▸; Fujioka & Takata, 1981 ▸).

A typical SAHase protomer is usually divided into three closely connected domains: an N-terminal substrate-binding domain, a cofactor-binding domain and a small C-terminal dimerization domain (Brzezinski *et al.*, 2012 ▸). The substrate-binding and cofactor-binding domains are separated by a bipartite hinge (Manszewski *et al.*, 2015 ▸), allowing them to oscillate between two conformational states, closed and open, during the catalytic cycle. The former state is assumed when the domains are close together and is thought to be stabilized by a ligand molecule (substrate, product or inhibitor) bound in the active site (Hu *et al.*, 1999 ▸; Yin *et al.*, 2000 ▸) and an alkali-metal or ammonium cation coordinated in a metal-binding loop near the active site (Brzezinski *et al.*, 2012 ▸; Manszewski *et al.*, 2015 ▸). However, the closed conformation has also been observed for a ligand-free protein (Zheng *et al.*, 2015 ▸). In the open conformation, which is considered to be the characteristic state of the ligand-free enzyme, the principal domains are spatially separated, forming a distinct substrate-access channel leading to the active site. Access to the active site is then regulated by two side chains (His and Phe) forming a molecular gate (Reddy *et al.*, 2008 ▸; Manszewski *et al.*, 2015 ▸).

Several crystal structures of SAHases from different organisms are available in the Protein Data Bank (PDB; Berman *et al.*, 2000 ▸); for example, mammalian [*Homo sapiens*, PDB entries 1a7a (Turner *et al.*, 1998 ▸) and 1li4 (Yang *et al.*, 2003 ▸); *Rattus norvegicus*, PDB entries 1b3r (Hu *et al.*, 1999 ▸), 1k0u (Huang *et al.*, 2002 ▸), 1ky5 (Takata *et al.*, 2002 ▸) and 1d4f (Komoto *et al.*, 2000 ▸)], plant (*Lupinus luteus*, PDB entries 3ond, 3one and 3onf; Brzezinski *et al.*, 2012 ▸), bacterial (*Mycobacterium tuberculosis*, PDB entry 3ce6; Reddy *et al.*, 2008 ▸) and protozoan (*Plasomodium falciparum*, PDB entry 1v8b; Tanaka *et al.*, 2004 ▸).

In our previous studies of BeSAHase from *Bradyrhiziobum elkanii*, which is the nitrogen-fixing bacterial symbiont of soybean (PDB entry 4lvc; Manszewski *et al.*, 2015 ▸), we described the enzyme in complex with Ado molecules that were bound in three of the four subunits of the tetramer. In this complex, the Ado-bound subunits were found in the closed conformation stabilized by an ammonium cation bound in the metal-binding loop, while the ligand-free subunit was in an open conformation and without an ammonium cation. Moreover, the molecular gate (MG) leading to the active site, formed by residues His342 and Phe343, was found to be shut when Ado was bound in the active site and open in the ligand-free subunit. As a continuation of these studies, here we present four crystal structures of BeSAHase in complex with different ligand molecules, namely as a mixed 3:1 complex with adenosine (Ado) and cordycepin (Cord; 3′-deoxy­adenosine), in complexes with adenine (Ade) and with adenosine (Ado), and as a mixed 1:1 complex with 2′-deoxy­adenosine (2′-dAdo) and Ade. The set of ligands used in our study is the same as in the study of LlSAHase from *L. luteus* (Brzezinski *et al.*, 2012 ▸) to facilitate comparison of the active-site architecture and protein conformation. The presented crystal structures shed new light on the question of subunit conformation in relation to the ligand type and on the details of the MG mechanism, showing that, contrary to the tentative view held to date, no simple correlation exists. In addition, small-angle X-ray scattering (SAXS) measurements conclusively demonstrate that the crystallographic model of the BeSAHase tetramer is consistent with the protein structure in solution.

## 2. Materials and methods   

### 2.1. Purification   

The purification of BeSAHase was carried out as described previously (Manszewski *et al.*, 2015 ▸) only for the mixed Ado/Cord complex (procedure P1). When it became apparent that the above ligand-exchange procedure *via* incubation does not necessarily lead to a complex with the desired ligand (see §[Sec sec3.1.1]3.1.1), a modification of the procedure of Yuan *et al.* (1993 ▸) was implemented as a step before size-exclusion chromatography to exchange the cofactor and prepare ligand-free enzyme for the subsequent incubation reaction. In this modified approach (procedure P2), 5 ml BeSAHase in buffer *A* (50 m*M* Tris pH 8.0, 500 m*M* NaCl, 20 m*M* imidazole) at a concentration of 12 mg ml^−1^ (estimated according to Bradford, 1976 ▸) was mixed with 10 ml saturated ammonium sulfate solution pH 3.3 and stored on ice for 10 min. The mixture was centrifuged, and the precipitate was dissolved in 5 ml buffer *A * and mixed again with ammonium sulfate solution as above. The pellet was again dissolved in 5 ml buffer *A* and mixed with 10 ml saturated ammonium sulfate solution pH 7.0. Finally, the pellet was dissolved in 5 ml buffer *A* and the protein concentration was adjusted to 8 mg ml^−1^. Ultimately, NAD^+^ was added in a 12-fold molar excess and the mixture was stored at 4°C for 12 h.

### 2.2. Crystallization   

#### 2.2.1. BeSAHase complex with adenosine/cordycepin   

BeSAHase solution obtained without the cofactor-exchange step (procedure P1) at a concentration of 12 mg ml^−1^ was incubated with an eightfold molar excess of cordycepin for 24 h. The mixture was submitted for crystallization in the High-Throughput Crystallization Facility at EMBL Hamburg (Mueller-Dieckmann, 2006 ▸) using vapour diffusion in sitting drops at 292 K. Single crystals suitable for X-ray diffraction experiments were obtained in 0.2 *M* sodium acetate, 16% PEG 4000, 0.1 *M* Tris pH 8.5 in two weeks.

#### 2.2.2. BeSAHase complexes with adenine, adenosine and adenine/2′-deoxyadenosine   

BeSAHase solution obtained with the additional purification step (procedure P2) described above was concentrated to 12 mg ml^−1^ and divided into three samples. Each sample was incubated for 24 h with an eightfold molar excess of Ade, Ado or 2′-dAdo, respectively, and crystallized by vapour diffusion in sitting drops at 292 K. Crystals of the Ade complex were obtained in condition No. 14 of the Morpheus crystal screen (Molecular Dimensions) consisting of 90 m*M* Halogens Mix (sodium fluoride, chloride and bromide), 40% ethylene glycol, 20% PEG 8000, 0.1 *M* imidazole, 0.1 *M* MES pH 6.5. Crystals of the Ado complex were obtained in 0.3 *M* sodium acetate, 16% PEG 4000, 0.1 *M* Tris pH 9.0. The third sample crystallized as an Ade/2′-dAdo complex using 0.3 *M* sodium acetate, 14% PEG 4000, 0.1 *M* Tris pH 8.0.

### 2.3. Data collection, structure solution and refinement   

Low-temperature X-ray diffraction data were collected on BESSY beamline 14.2 for the complexes with Ade and Ado, both to a resolution of 1.95 Å. Low-temperature X-ray diffraction data for the Ado/Cord and Ade/2′-dAdo complexes were collected on beamlines P14 and P13 at PETRA III, EMBL/DESY, Hamburg to resolutions of 1.84 and 1.54 Å, respectively. All crystals belonged to the orthorhombic system, space group *P*2_1_2_1_2. The diffraction data were processed and scaled with the *XDS* system (Kabsch, 2010 ▸). Data-collection statistics are presented in Table 1 ▸.

All structures were solved by molecular replacement with *Phaser* (McCoy *et al.*, 2007 ▸) using chain *A* of PDB entry 4lvc for BeSAHase (Manszewski *et al.*, 2015 ▸) as a model. Three of the four structures are roughly isomorphous and contain the complete BeSAHase tetramer in the asymmetric unit. The asymmetric unit of the Ade complex consists of a dimeric assembly and the complete tetramer is created by the crystallographic twofold axis along *z*. The *PHENIX* package (Adams *et al.*, 2010 ▸) was used for refinement of all of the structural models with maximum-likelihood targets and with TLS parameters assigned to rigid-body segments predicted by the *TLSMD* server (Painter & Merritt, 2006 ▸). *Coot* (Emsley & Cowtan, 2004 ▸) was used for manual model rebuilding between rounds of automatic refinement, as well as for building the ligand molecules and for validation of the solvent structure. The final structure-refinement statistics are shown in Table 1 ▸.

The Engh & Huber (2001 ▸) parameters were taken as stereochemical restraints for the protein chains and *elBOW* (Moriarty *et al.*, 2009 ▸), as implemented in *PHENIX* (Adams *et al.*, 2010 ▸), was used to create restraint libraries for all of the active-site ligands and cofactor molecules. All structures were standardized with *ACHESYM* (Kowiel *et al.*, 2014 ▸).

Atomic coordinates and structure factors have been deposited in the Protein Data Bank (PDB) with accession codes 5m5k (Ado/Cord complex), 5m65 (Ade), 5m66 (Ado) and 5m67 (2′-dAdo/Ade). Raw diffraction images were deposited in the RepOD repository (ICM, University of Warsaw) with the following DOIs: https://doi.org/10.18150/repod.1236363 (Ado/Cord), https://doi.org/10.18150/repod.7716153 (Ade), https://doi.org/10.18150/repod.8491539 (Ado) and https://doi.org/10.18150/repod.3824734 (2′-dAdo/Ade).

### 2.4. SAXS studies   

Small-angle X-ray scattering (SAXS) patterns were collected on beamline P12 at the PETRA III storage ring at EMBL/DESY, Hamburg. 20 µl of the protein sample at a concentration of 1.0, 2.0 and 4.0 mg ml^−1^ and 20 µl of the corresponding matching buffer were loaded into a 96-well plate. Automated loading of the SAXS samples into the cuvette was achieved using a Hamilton syringe robot (Hura *et al.*, 2009 ▸). SAXS data were collected over an *s* range of 0.0088–5.0 nm^−1^ and overlays of the merged data sets were used to detect concentration-dependent scattering in the lowest *s* region. The *ATSAS* package (Konarev *et al.*, 2006 ▸) was used to process all SAXS data. Integration, scaling and buffer subtraction were accomplished using *PRIMUS* (Konarev *et al.*, 2003 ▸). The resulting scattering curves were used for all calculations and reconstructions. The structural parameters of the protein were calculated using the *PRIMUS* package. The radius of gyration (*R*_g_) was computed using the Guinier approximation and the distance distribution function *p*(*r*) was calculated using the indirect Fourier transformation method as implemented in *GNOM* (Svergun, 1992 ▸). *CRYSOL* (Svergun *et al.*, 1995 ▸) was applied for evaluation of the solution scattering patterns using the crystallographic model of BeSAHase in complex with Ado. *Ab initio* modelling with fourfold symmetry was performed with *DAMMIN* (Svergun, 1999 ▸). The molecular weight of the analyzed protein was calculated from the extrapolated *I*(0) values in comparison to a standard protein (bovine serum albumin) sample using the equation
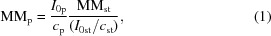
where MM_p_ and MM_st_ are the molecular weights of the studied and standard proteins, *I*_0p_ and *I*_0st_ are the scattering intensities at zero angle of the studied and standard protein solutions, respectively, and *c*_p_ and *c*_st_ are the concentrations of studied and standard protein, respectively. The SAXS data-collection statistics are presented in Table 2 ▸.

## 3. Results and discussion   

### 3.1. Overall structure of BeSAHase   

**Figure 1:**
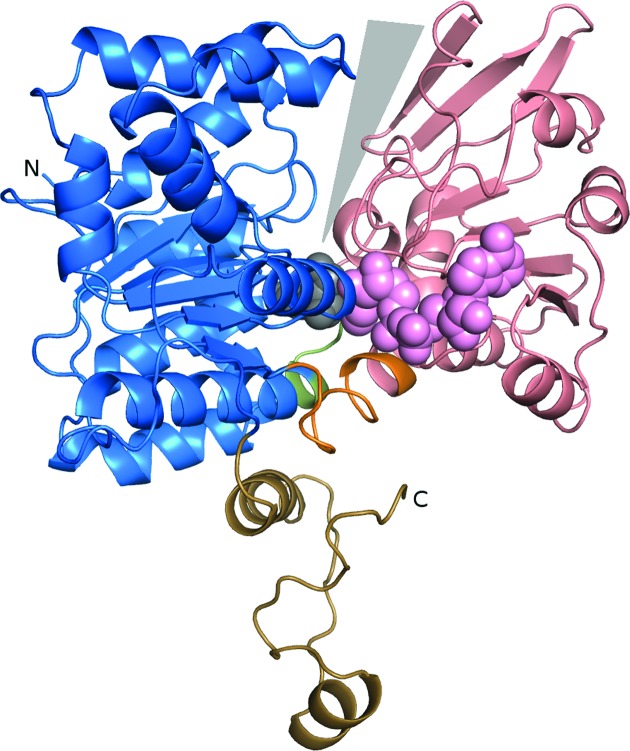
Subunit of BeSAHase in the closed conformation complexed with 2′-­deoxyadenosine. The domains are colour-coded as follows: blue, substrate-binding domain; salmon, cofactor-binding domain; sand, C-­terminal dimerization domain. The interdomain hinge regions are highlighted in orange (Asn222–Leu233) and green (His392–Val396). The grey triangle highlights the active-site access channel. The molecules of NAD^+^ (pink) and 2′-deoxyadeosine (grey) are shown in space-filling representation.

The overall structure of BeSAHase has been described in detail in our earlier paper (Manszewski *et al.*, 2015 ▸). Briefly, each subunit contains the native sequence of 473 amino-acid residues. The recombinant protein has six extra artifactual (GIDPFT–) residues at the N-terminus introduced by the cloning vector, but they are not visible in the electron-density maps of any of the models. BeSAHase protomers are divided into three domains, as illustrated in Fig. 1 ▸: a substrate-binding domain composed of residues Met1–Val221 and Met397–Val426, a cofactor-binding domain composed of residues Tyr234–Gly391, and a small C-terminal dimerization domain composed of residues Leu427–Tyr473. The first two domains are joined by two hinge regions comprised of residues Asn222–Leu233 and His392–Val396. It is notable that both hinge elements (demarcated according to main-chain torsions upon open–closed transition) contain α-helical turns of both conjoined subunits.

As described in §[Sec sec2.2]2.2, two types of protein preparations were used for the cocrystallization experiments. In one case (P2) the protein was precipitated with ammonium sulfate to remove any bound ligands prior to size-exclusion chromatography, and was then incubated with the desired cofactor (NAD^+^) and target ligands (adenosine or adenine or 2′-deoxyadenosine). In the other scenario (P1) no ligand removal (ammonium sulfate treatment) was used and the protein for the crystallization experiment was directly incubated with cordycepin.

All of the ligand and cofactor molecules in the presented complexes were modelled at full occupancy.

#### 3.1.1. Adenosine/cordycepin complex   

The structure of BeSAHase in the crystal obtained by cocrystallization with cordycepin of a protein sample that had not been treated with ammonium sulfate (see §[Sec sec2.2.1]2.2.1) is a tetramer with three sub­units (*A*, *B* and *C*) complexed with Ado molecules and one subunit (*D*) with Cord bound in the active site. The first five residues (MNAKP) of the genuine BeSAHase sequence of chains *A*, *B* anad *D* and two residues of chain *C* could not be modelled in electron density because of disorder. One NAD^+^ molecule is clearly defined by electron density in each subunit. There are three sodium cations near the active site of subunits *A*, *B* and *C*. 1490 water molecules plus three PEG molecules and one acetate ion from the crystallization buffer were located in the solvent region.

#### 3.1.2. Adenine complex   

The incubation of ligand-free BeSAHase (§[Sec sec2.2.2]2.2.2) with Ade resulted in crystals with a dimer in the asymmetric unit, from which the complete tetramer is created by crystallographic symmetry. The first four residues of chain *A* and the first five residues of chain *B* are not visible in electron density. In each subunit, one Ade molecule is found in the active site, accompanied by a sodium cation. Also, one NAD^+^ cofactor molecule is found in each protomer. Moreover, 570 water molecules, plus two bromide anions and five ethylene glycol molecules from the crystallization buffer, were modelled in the electron density of the solvent region.

#### 3.1.3. Adenosine complex   

Ligand-free BeSAHase incubated with Ado yielded crystals with a tetrameric assembly in the asymmetric unit. In each chain, the first five residues are not present in electron density. Moreover, residues Asn415–Ser418 of chain *D* and Asn415–Lys419 of chain *C* could not be modelled because of poor electron density. All four subunits have an Ado molecule bound in the active site. Also, four sodium cations are found in the metal-binding loop nearby. High-quality electron density defines one NAD^+^ molecule in each subunit. In addition, 896 water molecules and five PEG molecules from the crystallization buffer were found in the structure.

#### 3.1.4. Adenine/2′-deoxyadeosine complex   

Ligand-free BeSAHase incubated with 2′-deoxyadenosine gave the best diffracting crystals (1.54 Å resolution) and the structure revealed that the tetramer is composed of two subunits (*C* and *D*) complexed with the nucleoside used for incubation (2′-dAdo) and of two subunits (*A* and *B*) with adenine (Ade), which is a product of 2′-dAdo hydrolysis (Abeles *et al.*, 1980 ▸, 1982 ▸), in the active site. The first five genuine residues (MNAKP) of chains *A*, *B* and *C* and the first three residues of chain *D* could not be modelled because of disorder. Additionally, residues Asn415–Lys419 in chain *B* are not visible in electron density. As in the above structures, the NAD^+^ cofactor molecule was found in each protomer. 2156 water molecules, five sodium cations, three PEG molecules and two acetate ions were modelled in the structure.

### 3.2. The ligand nucleosides and adenine molecules   

**Figure 2:**
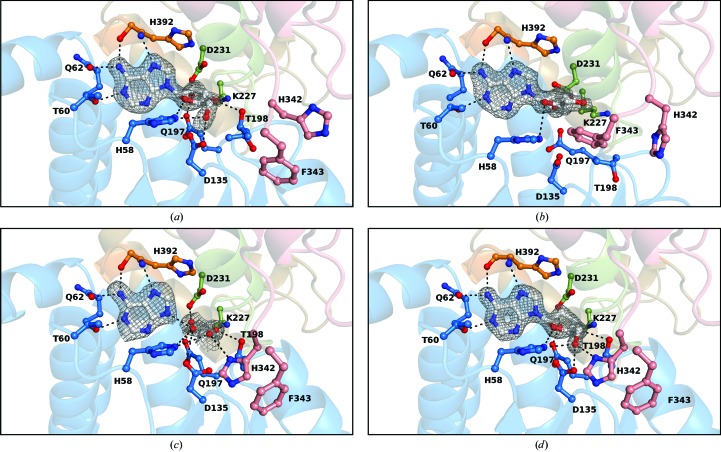
Comparison of BeSAHase active sites: (*a*) the Ado-complexed subunit *A* from the Ado/Cord complex, (*b*) the Cord-complexed subunit *D* from the Ado/Cord complex, (*c*) the Ade-complexed subunit *A* from the Ade complex and (*d*) the 2′-dAdo-complexed subunit *C* from the Ade/2′-dAdo complex. Since the mode of binding of all Ado molecules in all respective complexes is identical, only the ligand from the Ado/Cord structure is shown. Similarly, as the mode of binding of all Ade ligands is identical, only Ade from the Ade complex is shown. The ligands and water molecules are shown in *F*_o_ − *F*_c_ OMIT electron-density (calculated without the contribution of the nucleosides, Ade and water atoms to *F*_c_). The maps are contoured at 4σ, 3σ, 3σ and 4σ, respectively.

BeSAHase was incubated and crystallized with four different ligands: cordycepin (3′-deoxyadenosine), adenine, adenosine and 2′-deoxyadenosine. The latter three incubations were preceded by precipitation with ammonium sulfate to remove all ligands bound in the overexpression step. In each structure, the ligand molecules used for incubation were unambiguously identified (at least in some subunits) in difference electron density (Fig. 2 ▸). However, in two cases the desired ligands were found only in some subunits, whereas the remaining subunits were occupied either by a competing ligand from the expression system (Ado) or by a product of ligand hydrolysis (Ade). The polar interactions between the protein and the ligand molecules are listed in Table 3 ▸. In all structures the adenine moiety is bound in the same mode. All N atoms except N3 form hydrogen bonds with both main-chain and side-chain atoms of BeSAHase. In the case of the sugar moieties, however, there are differences in the hydrogen-bond networks resulting from the different ribose O-atom patterns as well as from the protein conformation. The conformational details of all of the nucleoside ligands, including the pseudorotation parameters of the sugar rings, are listed in Table 4 ▸.

#### 3.2.1. Adenosine   

Ado molecules were found in two of the four structures presented here. In the Ado/Cord complex, which was obtained using protein untreated with ammonium sulfate and incubated with cordycepin, it is serendipitously found (Fig. 2 ▸ *a*) in three of the four subunits. This indicates that the adenosine molecules were sequestered by the protein in the overexpression step and retained during purification. Since the protein:cordycepin molar ratio was 1:8 and the original Ado ligand was apparently replaced in only 25% of the cases, this may suggest that the affinity of BeSAHase for cordycepin is over an order of magnitude lower than that for adenosine, leading to the conclusion that cordycepin would not be a good candidate as an inhibitor of SAHase. However, inferences about complex-formation equilibria in solution from crystallization effects should be regarded with caution.

The intended Ado complex was obtained from ligand-free protein incubated with an eightfold molar excess of the nucleoside. In this case, all four subunits have the active site occupied by Ado.

The interactions between the Ado molecules and the protein in these two complexes are the same and are similar to those described previously (Manszewski *et al.*, 2015 ▸). There is, however, one important difference: in the present complexes the side chain of His342, which is an element of the molecular gate (see §[Sec sec3.3.1]3.3.1), is shifted away from the active site and the hydrogen bond between the His342 N^δ1^ atom and the O5′ atom of Ado is not formed. The sugar rings have an O4′-*endo*-C4′-*exo* pucker in Ado from the Ado/Cord complex and a C2′-*endo* pucker in Ado molecules from the Ado complex.

#### 3.2.2. Cordycepin   

A cordycepin molecule was found in subunit *D* of the Ado/Cord complex (Fig. 2 ▸ *b*). Owing to the conformational changes of this subunit when compared with subunits *A*, *B* and *C*, and to the absence of the ribose O3′ atom, the hydrogen-bond network around the cordycepin ligand is significantly different. Specifically, the hydrogen bonds between O3′ and the Thr198 O^γ1^ and Lys227 N^ζ^ atoms that are observed in the Ado-occupied subunits are absent. The backbone conformation of subunit *D* is sufficiently different (see §[Sec sec3.3]3.3) as to place the side chain of Glu197 in a different direction, so that the hydrogen bond between the O2′ atom of cordycepin and Glu197 O^∊2^ cannot be formed (the distance between these atoms is 4.1 Å). Also, the hydrogen bonds created with the participation of the O5′ atom are not observed in chain *D* of the Ado/Cord complex , because the γ torsion angle (defined by the O5′—C5′—C4′—C3′ atoms of the ribose moiety; Table 4 ▸) of the cordycepin ligand is twisted by ∼100° with respect to that of the Ado molecules. The Cord sugar ring puckering is C4′-*exo*, which is in good agreement with the puckering of the ribose moiety of cordycepin bound to plant SAHase (LlSAHase from *L. luteus*; Brzezinski *et al.*, 2012 ▸; PDB entry 3onf).

#### 3.2.3. Adenine   

Ade molecules were found in two structures. In the complex formed by the incubation of BeSAHase with Ade, all subunits have this ligand bound in the active site (Fig. 2 ▸ *c*). In the Ade/2′-dAdo complex, two (*A* and *B*) of the four subunits are complexed with Ade, which is a product of 2′-deoxyadenosine hydrolysis (Abeles *et al.*, 1980 ▸, 1982 ▸). In both structures, despite the absence of the sugar moiety, the architecture of the active site is preserved, as observed previously by Brzezinski *et al.* (2012 ▸). In a pattern of molecular mimicry, four water molecules imitate the positions of the O2′, O3′, O4′ and O5′ atoms of an Ado molecule and create hydrogen bonds to the corresponding side-chain atoms of the protein. Since there are seven different Ado molecules among the structures reported here that can be used as a reference for the ribose-mimicking water molecules in the Ade complexes, the deviations in atom positions of the water molecules in each Ade complex have been averaged over the Ado templates. In each case the reported values are given for O2′, O3′, O4′ and O5′, respectively. For the water molecules in subunits *A* and *B* of the Ade complex these differences are 0.23 and 0.32, 0.26 and 0.20, 0.77 and 1.01, and 1.08 and 1.11 Å, respectively. The second model that contains Ade molecules is the complex obtained by cocrystallization with 2′-dAdo. The Ade molecules are bound in subunits *A* and *B* and the corresponding water molecules are superposable with Ado ribose O atoms within 0.65 and 0.69, 0.39 and 0.29, 0.45 and 0.34, and 0.21 and 0.39 Å, respectively. It is of note that one of these water molecules always replaces the O2′ atom, even though no such atom is present in the 2′-dAdo ligand of the Ade/2′-dAdo complex. The hydrogen bonds created by these water molecules are the same in each case, although their distances vary within ∼0.4–0.6 Å. This is because the positions of the corresponding water molecules differ, on average, by 0.55, 0.33, 0.59 and 0.87 Å for the molecules that mimic the positions of O2′, O3′, O4′ and O5′, respectively.

In contrast to the protein–nucleoside interactions of the abovementioned complexes, in the Ade complexes there is an additional hydrogen bond between His342 N^δ1^ and the water molecule that mimics the O5′ position. A similar His342 N^δ1^⋯O5′ interaction was found in the Ado complex of BeSAHase reported previously (Manszewski *et al.*, 2015 ▸; PDB entry 4lvc). In the Ade/2′-dAdo complex there is also an additional water molecule in the Ade-complexed subunits that is superposable within 0.41 Å with the C1′ atom of the 2′-dAdo molecules and does not interact with any protein atoms.

#### 3.2.4. 2′-Deoxyadeosine   

In the Ade/2′-dAdo complex, subunits *C* and *D* have 2′-dAdo in the active site (Fig. 2 ▸ *d*). A comparison of these subunits with those complexed with Ade (chains *A* and *B*) reveals that the geometry of the hydrogen-bond networks between the ligand and protein atoms is nearly the same. Superposition of a 2′-dAdo molecule on Ade shows that all of the O atoms of 2′-dAdo overlap with water molecules within 0.19 Å (O3′), 0.47 Å (O4′) and 0.26 Å (O5′). Moreover, another water molecule is present in the 2′-dAdo-complexed subunits, which mimics the position of the O2′ atom of an Ado molecule and creates two hydrogen bonds, with Glu197 O^∊2^ and Asp231 O^δ2^, that are characteristic of the Ado complexes.

### 3.3. Ligand type *versus* protein conformation   

A simplified view holds that the conformation of SAHase subunits switches from open to closed upon ligand binding (Hu *et al.*, 1999 ▸), although it was shown by Zheng *et al.* (2015 ▸) that the closed conformation can also be maintained without ligand molecules in the active site. In our previous BeSAHase structure (Manszewski *et al.*, 2015 ▸), the protein was found in two different conformational states within one tetramer: the three Ado-complexed subunits were in the closed conformation, while one ligand-free subunit was open.

Analysis of subunit conformation in the present structures, carried out by means of r.m.s.d. calculations for superposed C^α^ atoms (Table 5 ▸), reveals that, except for the subunit complexed with cordycepin, all other BeSAHase subunits are in the closed conformation. The r.m.s.d. values between these subunits and a closed subunit from the earlier BeSAHase model with PDB code 4lvc (chain *A*; Manszewski *et al.*, 2015 ▸) range from 0.16 Å for an Ade-complexed subunit of the Ade/2′-dAdo complex to 0.31 Å for the Ade complex. The most interesting situation is found in subunit *D* of the Ado/Cord complex, where cordycepin is bound. The r.m.s.d. values between this subunit and the closed/open subunits of the PDB entry 4lvc are 1.71 and 1.29 Å, respectively. This shows that BeSAHase in complex with cordycepin adopts an semi-open conformation that is halfway between the closed and open forms. This is in contrast to the cordycepin complex of LlSAHase (Brzezinski *et al.*, 2012 ▸), which is much closer to the standard closed form (0.44 Å) than to the Cord-complexed subunit of BeSAHase (1.66 Å).

#### 3.3.1. The molecular gate   

**Figure 3:**
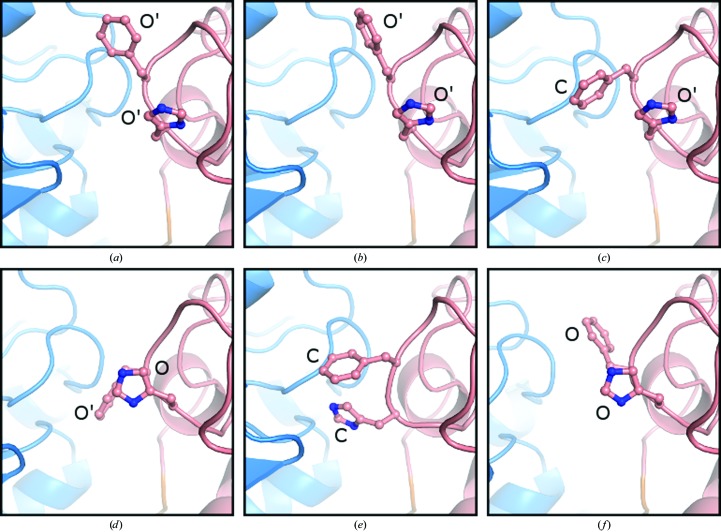
Comparison of the side-chain conformations of the MG residues in (*a*) the Ado-complexed subunit of the Ado/Cord complex, (*b*) subunit *A* of the Ado complex, (*c*) subunit *B* of the Ado complex, (*d*) the Cord-complexed subunit of the Ado/Cord complex, (*e*) subunit *A* of the Ade complex and (*f*) the open subunit (*D*) of PDB entry 4lvc. The side chains of the molecular-gate residues His342 and Phe343 are shown in ball-and-stick representation and annotated as C (closed state), O (open state) or O′ (variant open state; see text).

In the structure of *M. tuberculosis* SAHase (Reddy *et al.*, 2008 ▸), the side chain of His363 (which corresponds to His342 in BeSAHase) was recognized as a ‘molecular gate’ (MG) that opens or shuts an access channel leading to the active site. In the structure of LlSAHase (Brzezinski *et al.*, 2012 ▸) the MG was shown to actually consist of a tandem of His-Phe residues. Also, in the structure of BeSAHase with PDB code 4lvc (Manszewski *et al.*, 2015 ▸) the MG element was shown to consist of His342-Phe343 and appeared to be in the open/shut state in correlation with the open/closed conformation of the protein chain. This simple rule is not preserved in the present structures as the MG state varies even among subunits complexed with the same ligand (Fig. 3 ▸).

In the Ado-bound (closed) subunits *A*, *B* and *C* of the Ado/Cord complex (Fig. 3 ▸ *a*), the side chains of the MG element are in an open conformation; that is, in a different state to that in the Ado-bound closed subunits of the 4lvc structure (Manszewski *et al.*, 2015 ▸). The same situation is found in chains *A* and *D* of the Ado complex (Fig. 3 ▸ *b*). In subunits *B* and *C* of the same complex (Fig. 3 ▸ *c*), the side chain of His342 remains in the open state (and for this reason there is no hydrogen bond between the Ado O5′ atom and His342 N^δ1^; see §[Sec sec3.2.1]3.2.1), but the side chain of Phe343 assumes a conformation that is compatible with the shut state of the MG. In the Cord-complexed subunit the side chain of His342 adopts exactly the same conformation as in the open subunit of BeSAHase 4lvc, but the side chain of Phe343, while remaining in the open conformation, is rotated by ∼180° around the C^α^—C^β^ bond (Fig. 3 ▸ *d*). The MG residues in all the Ade- and 2′-dAdo-complexed subunits (Fig. 3 ▸ *e*) are in the shut conformation, which allows the formation of the Ado O5′⋯N^δ1^ His342 hydrogen bond.

### 3.4. Sodium cations   

**Figure 4:**
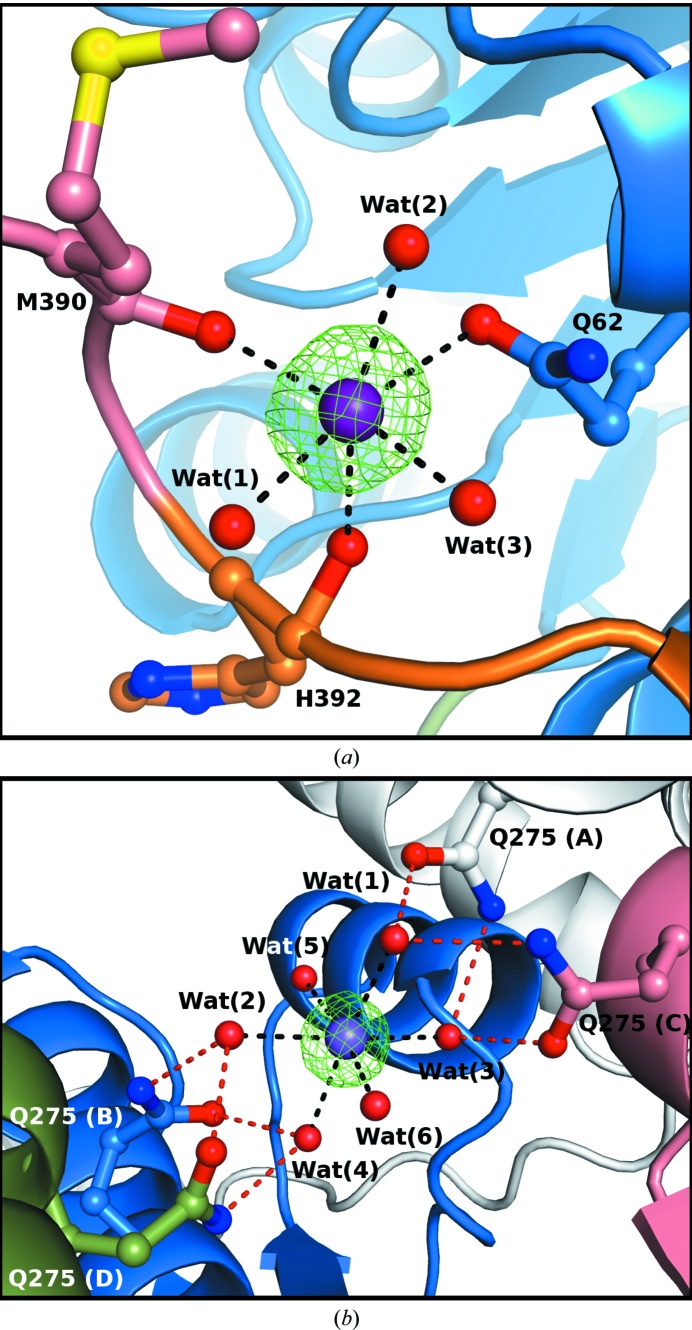
Coordination of sodium cations (purple spheres). (*a*) The Na^+^ cation bound in subunit *A* of the Ade/2′-dAdo complex. The coordination bond (dashed lines) distances are listed in Table 6 ▸. (*b*) The Na^+^ cation bound in the core of the Ade/2′-dAdo complex (the Na–Wat coordination-bond distances are listed in Table 7 ▸). Hydrogen bonds are represented by red dashed lines. In both panels, the *F*_o_ − *F*_c_ OMIT electron density was calculated without the contribution of Na^+^ to *F*_c_ and contoured at 5σ. The domains are colour-coded as in Fig. 1 ▸. Water molecules are represented as red spheres.

In all structures presented here there are sodium cations identified in clear electron density (Fig. 4 ▸). The metal identification was confirmed by the *B* factors (similar to those of the coordinating ligand atoms) and by the coordination geometry (Tables 6 ▸ and 7 ▸), and was additionally validated using the *CheckMyMetal* (*CMM*) server (Zheng *et al.*, 2014 ▸). Except for the Cord-complexed subunit, which is metal-free, the Na^+^ ions are present in the Ala389–Pro393 loop, which is located near the active site. Additionally, a sodium ion was found in the core of the Ade/2′-dAdo complex near the interface of all four subunits.

#### 3.4.1. Sodium cations near the active site   

In the structure of BeSAHase with the PDB code 4lvc (Manszewski *et al.*, 2015 ▸), an ammonium cation was modelled in the electron density in the loop Ala389–Pro393 that overlaps the His392–Val396 element of the interdomain hinge. The NH_4_^+^ ion was hydrogen-bonded by three water molecules and the carbonyl O atoms of Met390 and His392 (one of the NH donors was assumed to form a bifurcated hydrogen bond). The coordination sphere of the present sodium cations is comprised of the same five ligands as in the case of the NH_4_^+^ cation but is complemented into an octahedral pattern by the Gln62 O^∊1^ atom (Fig. 4 ▸ *a*, Table 6 ▸). The absence of the sodium cation in the Cord-complexed subunit, which adopts an intermediate open/closed conformation, and the fact that BeSAHase in the open conformation also does not bind any ion (Manszewski *et al.*, 2015 ▸), suggest that cation binding in the Ala389–Pro393 loop rigidifies the interdomain hinge and stabilizes the protein in the closed conformation.

#### 3.4.2. A sodium cation in the core of the adenine/2′-deoxyadeosine complex   

Careful analysis of the electron-density maps for the highest resolution (1.54 Å) Ade/2′-dAdo complex structure revealed an additional sodium cation located right in the centre of the BeSAHase tetramer (Fig. 4 ▸ *b*), where amino-acid residues from the cofactor-binding domains of all four subunits meet. The slightly distorted octahedral coordination sphere is created by six water molecules, four of which are hydrogen-bonded by the Gln275 O^∊1^ and N^∊2^ atoms. Since the interaction interfaces between the subunits of the BeSAHase tetramer, as calculated with *PDBsum* (Laskowski, 2009 ▸) and reported by Manszewski *et al.* (2015 ▸), are relatively large (∼2900 Å^2^ between subunits forming a dimer and ∼1600 Å^2^ between juxtaposed subunits), the binding of the core sodium cation appears to be artifactual as it does not seem to be important for tetramer stabilization.

### 3.5. Cofactor molecules   

For its enzymatic activity, SAHases requires one molecule of nicotinamide adenine dinucleotide in its oxidized form (NAD^+^) bound near the active site of each subunit. The mode of NAD^+^ binding by BeSAHase has been described in detail in our previous work (Manszewski *et al.*, 2015 ▸). Briefly, residues of both the cofactor-binding and substrate-binding domains take part in the hydrogen-bond network formed between the protein and cofactor atoms. Moreover, the side chains of residues Lys467 and Tyr471 from the C-terminal domain from an adjacent subunit are involved in cofactor binding, highlighting the role of the C-terminal domain in the dimerization process.

**Figure 5:**
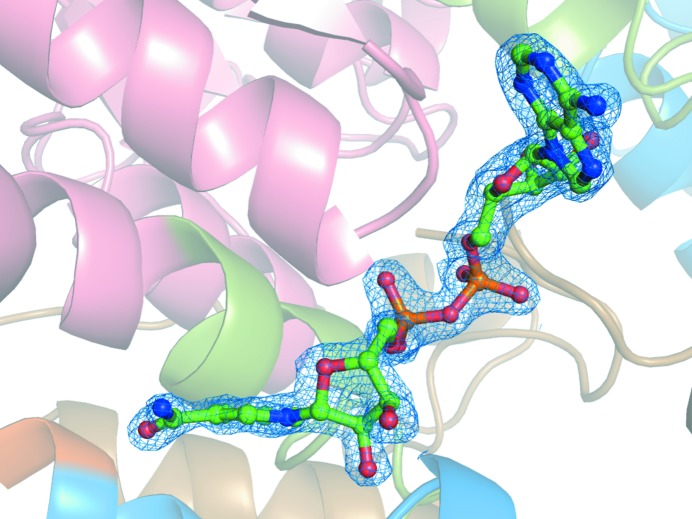
The NAD^+^ cofactor molecule bound in subunit *A* of the Ade/2′-dAdo complex, shown in 2*F*_o_ − *F*_c_ electron density contoured at 1σ. The flat electron density of the nicotine ring confirms the oxidized state of the cofactor.

In the structures reported here, the cofactor was supplied to the protein using two different procedures (P1 and P2; see §[Sec sec2.1]2.1). Notwithstanding this difference, in each structure the cofactor was found in the oxidized (NAD^+^) rather than the reduced (NADH) form. The main piece of evidence for the cofactor oxidation state is the electron density of the nicotine moiety clearly indicating a planar ring. Meijers *et al.* (2001 ▸) showed that the addition of the hydride ion to the nicotine ring in the oxidized form leads to loss of aromatic character and geometrical deformation into a boat conformation, which are characteristic of the reduced (NADH) form. In the present study, the cofactor molecules were refined without planarity restraints to probe the geometry of the nicotine ring. Despite the less-than-atomic resolution of the diffraction data, the refinement in each case was stable and converged with a flat nicotine ring, as illustrated in Fig. 5 ▸ for the NAD^+^ molecule from subunit *A* of the Ade/2′-dAdo complex.

### 3.6. SAXS studies   

The global structure of BeSAHase in solution was reconstructed using small-angle X-ray scattering (SAXS) in order to confirm the oligomerization state in solution. SAXS data inform about the overall size and shape of macromolecules in solution and can be used to generate a global structural model at resolutions of about 12 Å or above.

The radius of gyration (*R*_g_) of BeSAHase calculated from the Guinier approximation is largely independent of concentration: 38.3 Å at 4 mg ml^−1^ protein concentration, 39.3 Å at 2 mg ml^−1^ and 39.8 Å at 1 mg ml^−1^ (see Supplementary Fig. S1). The theoretical value of *R*_g_, calculated using the coordinates of the BeSAHase tetramer from the crystal structure of the Ado complex, is 36 Å, while the theoretical values calculated for the monomer and dimer are 22.5 and 30 Å, respectively, confirming that BeSAHase is tetrameric in solution. The presence of a tetramer in solution is confirmed by the χ^2^ parameters calculated in *CRYSOL*, which are 753.2, 482.7 and 8.78 for the momoner, dimer and tetramer, respectively.

**Figure 6:**
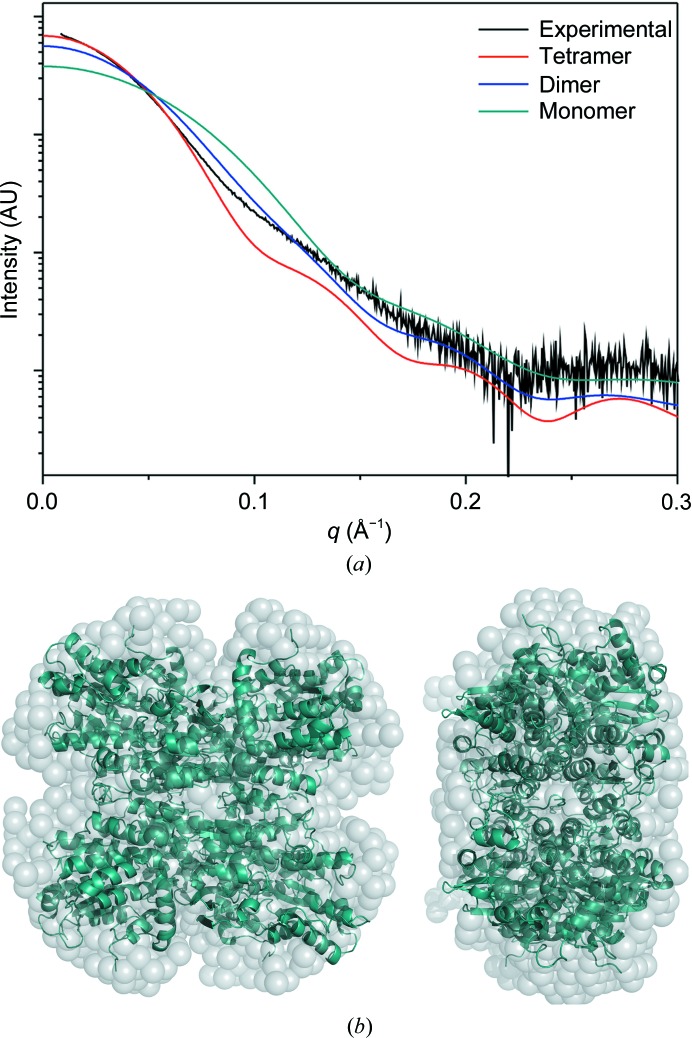
(*a*) Comparison of the experimental SAXS curve for BeSAHase (collected at a protein concentration of 4 mg ml^−1^) with theoretical SAXS scattering curves calculated for the tetramer, dimer and monomer using the crystallographic coordinates from the present study. (*b*) Molecular envelope of BeSAHase obtained with *DAMMIN* (Svergun, 1999 ▸), with the crystallographic model of the Ado complex superposed. The two views are related by a 90° rotation around the vertical axis in the plane of the drawing.

The experimental and theoretical SAXS curves are presented in Fig. 6 ▸(*a*). A comparison of the crystallographic structure with an *ab initio* model derived from the SAXS data, shown in Fig. 6 ▸(*b*), additionally corroborates the tetrameric quaternary state of the protein in solution.

## 4. Conclusions   

Four crystal structures of *S*-adenosyl-l-homocysteine hydrolase from *B. elkanii* were solved in complex with different ligands, (i) mixed adenosine/cordycepin, (ii) adenine, (iii) adenosine and (iv) mixed adenine/2′-deoxyadenosine, to resolutions of 1.84, 1.95, 1.95 and 1.54 Å, respectively. The Ado molecules in structure (i) were sequestered by the protein during overexpression in *E. coli* cells. All other ligands [except for Ade, which in (iv) is a product of hydrolysis of 2′-dAdo by BeSAHase] were added to the protein solution after the purification step, which included a careful ligand-removal and cofactor-exchange procedure. The NAD^+^ cofactor is found at full occupancy in the cofactor-binding domain of all of the subunits and also in case (i), where no cofactor exchange was applied. The various active-site ligands were all modelled at full occupancy in unambiguous electron-density maps, also in complexes (i) and (iv) where subsets of subunits of the tetrameric enzyme are charged with different cargo. The architecture of the active site is conserved, even when Ade, a ligand without a sugar ring moiety, is bound. This is because well defined water molecules mimic the pattern of the ribose O atoms in largely conserved hydrogen-bond interactions. The conformation of BeSAHase is affected by the ligand type in the active site and in all cases except for the cordycepin complex the subunit is closed. When cordycepin is bound in the active site, the protein molecule assumes a semi-open conformation that is intermediate between the canonical open and closed states. The side chains of His342 and Phe343 (both from the cofactor domain) form a molecular gate that controls passage through an active-site access channel formed between the substrate-binding and cofactor-binding domains. There is no apparent correlation between the ligand type and the open/shut state of the molecular gate, which can even vary with the same ligand molecule bound in the active site. There is also no unambiguous correlation between the open/closed conformation of the protein subunit and the open/shut state of the molecular gate. Moreover, the gate can also assume ‘ambiguous’ states, with one side chain in the open conformation and the other in the shut conformation. Certainly, further studies are needed to elucidate the actual mechanism of the molecular gate. A sodium cation is coordinated in a metal-binding loop near the active site of all subunits in the closed conformation, and this cation binding seems to be the most constant structural feature that can be correlated with the conformational state of the SAHase subunit. The metal-binding loop overlaps a hinge region between the substrate and cofactor domains. Metal (or ammonium) coordination in this region helps to fix the molecular conformation in the closed state. The identity of the metal ion was confirmed by the pattern of Na–O distances (including *CMM* tests), by the electron density and by successful refinement. Small-angle X-ray scattering at three different protein concentrations confirmed the tetrameric state of BeSAHase in solution.

## Supplementary Material

PDB reference: BeSAHase, complex with adenosine and cordycepin, 5m5k

PDB reference: complex with adenine, 5m65

PDB reference: complex with adenosine, 5m66

PDB reference: complex with 2′-deoxyadenosine and adenine, 5m67

Supporting Information: Supplementary Figure S1.. DOI: 10.1107/S2052252517002433/lz5013sup1.pdf

Raw X-ray diffraction data for complex of BeSAHase with adenosine and cordycepin. URL: https://doi.org/10.18150/repod.1236363

Raw X-ray diffraction data for complex of BeSAHase with adenine. URL: https://doi.org/10.18150/repod.7716153

Raw X-ray diffraction data for complex of BeSAHase with adenosine. URL: https://doi.org/10.18150/repod.8491539

Raw X-ray diffraction data for complex of BeSAHase with 2′-deoxyadenosine and adenine. URL: https://doi.org/10.18150/repod.3824734

## Figures and Tables

**Table 1:** Data-collection and refinement statistics Values in parentheses are for the last resolution shell.

Complex	Ado/Cord	Ade	Ado	Ade/2′-dAdo
Data collection				
Beamline	P14, EMBL/DESY	14.2, BESSY	14.2, BESSY	P13, EMBL/DESY
Wavelength (Å)	1.223	0.918	0.918	0.969
Temperature (K)	100	100	100	100
Space group	*P*2_1_2_1_2	*P*2_1_2_1_2	*P*2_1_2_1_2	*P*2_1_2_1_2
Unit-cell parameters				
*a* (Å)	108.2	100.7	106.4	107.5
*b* (Å)	176.3	103.1	173.6	174.5
*c* (Å)	102.3	90.7	97.4	96.6
Resolution (Å)	47.82–1.84 (1.95–1.84)	45.87–1.95 (2.06–1.95)	46.90–1.95 (2.07–1.95)	43.63–1.54 (1.63–1.54)
Total reflections	1276255	283603	481479	2486875
Unique reflections	168719	69284	130113	268793
Multiplicity	7.6 (5.1)	4.1 (4.1)	3.7 (3.7)	9.3 (9.1)
Completeness (%)	98.6 (91.7)	99.1 (97.3)	98.9 (96.0)	99.6 (98.3)
〈*I*/σ(*I*)〉	16.62 (2.01)	10.06 (2.17)	13.01 (1.93)	15.41 (2.19)
*R*_merge_ †	0.098 (1.005)	0.131 (0.739)	0.087 (0.740)	0.096 (1.021)
Refinement				
Working/test reflections	167476/1243	68276/1008	129104/1009	267784/1009
*R*/*R*_free_ ‡	0.148/0.192	0.171/0.209	0.170/0.209	0.145/0.176
No. of atoms				
Protein	14702	7291	14527	14680
Ligand	75	20	76	56
NAD^+^	176	88	176	176
Water	1490	570	896	2156
Na^+^ ions	3	2	4	5
Acetate ions	1	—	—	2
PEG molecules	3	—	5	3
Ethylene glycol	—	5	—	—
Br^−^ ions	—	2	—	—
〈*B*〉 factors (Å^2^)				
Protein	27.4	30.1	35.1	21.6
Ligand	21.4	31.8	27.4	16.5
NAD^+^	19.3	19.6	28.0	15.5
Na^+^ ions	25.0	36.9	27.1	16.4
Water	35.9	33.2	38.0	33.8
R.m.s.d. from ideality for bonds (Å)	0.012	0.017	0.016	0.015
Ramachandran statistics (%)				
Favoured	97.3	96.7	95.9	97.5
Outliers	2.7	3.3	4.1	2.5
PDB code	5m5k	5m65	5m66	5m67

† *R*_merge_ = 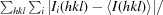 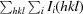, where 〈*I*(*hkl*)〉 is the average intensity of reflection *hkl*.

‡ *R *= 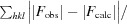 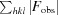, where *F*_obs_ and *F*_calc_ are the observed and calculated structure factors, respectively. *R*_free_ is calculated analogously for the test reflections, which were randomly selected and excluded from the refinement.

**Table 2:** SAXS data-collection and scattering-derived parameters

Data collection			
Instrument	P12, PETRA III	P12, PETRA III	P12, PETRA III
Wavelength (Å)	1.24	1.24	1.24
*q* range (nm^−1^)	0.0088–5	0.0088–5	0.0088–5
Exposure time (s)	1	1	1
Concentration (mg ml^−1^)	1	2	4
Temperature (K)	293	293	293
Structural parameters			
*I*(0) (arbitrary units) [from *P*(*r*)]	54.82 ± 1	62.37 ± 1	68.92 ± 1
*R*_g_ (Å) [from *P*(*r*)]	39.7	39.2	38.2
*I*(0) (arbitrary units) (from Guinier)	54.95 ± 1	62.45 ± 1	68.97 ± 1
*R*_g_ (Å) (from Guinier)	39.8	39.3	38.3
*D*_max_ (Å)	117	116	102.5
Porod volume estimate (Å^3^)	258549	258931	259165
Dry volume calculated from sequence (Å^3^)	251864	251864	251864
Molecular-mass determination			
Contrast (Δρ × 10^10^ cm^−2^)	3.047	3.047	3.047
Molecular mass *M*_r_ [from *I*(0)] (kDa)	220	220	220
Calculated monomeric *M*_r_ from sequence (kDa)	52.05	52.05	52.05
Software used			
Primary data reduction	*PRIMUS*		
Data processing	*PRIMUS*		
*Ab initio* analysis	*DAMMIN*		
Validation and averaging	*DAMAVER*		
Computation of model intensities	*CRYSOL*		
Three-dimensional graphics representation	*PyMOL*		

**Table 3:** Polar interactions between BeSAHase atoms and molecules in the active site Since the interactions of the same ligand molecules bound in the same complex are very similar, only values for the ligands bound in subunit *A* (Ado and Ade) or subunit *C* (2′-dAdo) are listed. Distances are in Å.

	Atom in											
	Ado/Cord complex								Ade/2′-dAdo complex			
	Ado		Cord		Ade complex		Ado complex		Ade		2′-dAdo	
Residue and atom	Atom	Distance	Atom	Distance	Atom	Distance	Atom	Distance	Atom	Distance	Atom	Distance
His58 N^∊2^	O4′	3.23	O4′	3.44	Wat3	3.46	O4′	2.95	Wat3	3.38	O4′	3.07
His58 N^∊2^	O5′	2.81	—	—	Wat4	3.38	O5′	2.66	Wat4	2.90	O5′	2.79
Thr60 O^γ1^	N1	2.67	N1	2.76	N1	2.77	N1	2.65	N1	2.72	N1	2.72
Gln62 O^∊1^	N6	2.91	N6	2.92	N6	2.87	N6	2.84	N6	2.86	N6	2.86
Asp135 O^δ1^	O5′	2.93	—	—	Wat4	3.21	O5′	2.66	Wat4	2.77	O5′	2.73
Glu197 O^∊2^	O2′	2.51	—	—	Wat1	2.77	O2′	3.12	Wat1	2.37	Wat1	2.33
Thr198 O^γ1^	O3′	3.14	—	—	Wat2	2.90	O3′	2.91	Wat2	3.05	O3′	3.00
Lys227 N^ζ^	O3′	2.97	—	—	Wat2	2.72	O3′	3.03	Wat2	2.96	O3′	2.94
Asp231 O^δ2^	O2′	2.55	O2′	2.47	Wat1	2.62	O2′	2.52	Wat1	2.61	Wat1	2.48
His342 N^δ1^	—	—	—	—	Wat4	3.39	—	—	Wat4	2.65	O5′	2.57
His392 N	N7	3.03	N7	2.89	N7	2.77	N7	2.86	N7	2.85	N7	2.88
His392 O	N6	3.01	N6	3.06	N6	2.93	N6	3.03	N6	3.03	N6	3.04

**Table 4:** Conformation (°) of the nucleoside molecules The torsion angles defining the nucleoside conformation are as follows: χ, C4—N9—C1′—O4′; γ, O5′—C5′—C4′—C3′; ν_0,_ C4′—O4′—C1′—C2′; ν_1_, O4′—C1′—C2′—C3′ *etc*. Since the conformation of the same ligand molecules bound in the same complex is very similar, only values for the ligands bound in subunit *A* (Ado and Ade) or subunit *C* (2′-dAdo) are listed. The amplitude (τ_m_) and the phase angle (*P*) of pseudorotation (with estimated standard deviations in parentheses, given in units of the last significant digit) were calculated by the method of Jaskólski (1984 ▸).

	Ado/Cord complex			
	Ado	Cord	Ado	2′-dAdo
Glycosidic bond χ	−106.95 (*anti*)	−112.78 (*anti*)	−103.30 (*anti*)	−98.71 (*anti*)
γ	−163.2	−58.1	−161.6	−165.3
ν_0_	−34.4	−25.2	−41.3	−40.6
ν_1_	13.8	5.0	25.5	35.9
ν_2_	10.1	15.9	−1.3	−18.3
ν_3_	−30.3	−32.3	−22.9	−5.2
ν_4_	41.0	35.9	41.0	30.4
*P*	75.4 (8)	62.7 (5)	163.9 (8)	116.8 (10)
τ_m_	40.4 (6)	35.0 (3)	42.7 (6)	41.1 (7)
Sugar pucker	O4′-*endo*-C4′-*exo* (^O^_4_ *T*)	C4′-*exo* (_4_ *T* ^O^)	C2′-*endo* (^2^ *E*)	C1′-*exo* (_1_ *T* ^O^)

**Table 5:** R.m.s. deviations (Å) for C^α^ atoms of superposed subunits of BeSAHase complexed with different ligands The closed and open subunits from the BeSAHase model with PDB code 4lvc (Manszewski *et al.*, 2015 ▸) are included for comparison. Since the values for the same ligand molecules bound in the same complex are very similar, only data for the ligands bound in subunit *A* (Ado and Ade) or subunit *C* (2′-dAdo) are listed. Values were calculated with *ALIGN* (Cohen, 1997 ▸).

Model†	**Ado**/Cord	Ado/**Cord**	Ade	Ado	Ade/**2′-dAdo**	**Ade**/2′-dAdo	4lvc closed
Ado/**Cord**	1.68						
Ade	0.30	1.54					
Ado	0.26	1.66	0.30				
Ade/**2′-dAdo**	0.20	1.69	0.32	0.22			
**Ade**/2′-dAdo	0.19	1.75	0.35	0.21	0.14		
4lvc closed	0.18	1.71	0.31	0.28	0.21	0.16	
4lvc open	2.32	1.29	2.17	2.27	2.33	2.38	2.32

† In mixed complexes, the subunit used for comparison is indicated by the ligand in bold.

**Table 6:** Details of sodium-ion coordination (Å) near the active site In each structure, the interactions are nearly identical in each subunit; therefore, only values for the ions bound in subunit *A* are listed.

Ligand atom	Ado/Cord complex	Ade complex	Ado complex	Ade/2′-dAdo complex
Gln62 O^∊1^	3.16	3.31	2.92	2.83
Met390 O	2.71	2.63	2.46	2.48
His392 O	2.74	3.18	2.68	2.73
Wat1	2.46	2.38	2.30	2.43
Wat2	2.80	2.72	2.53	2.54
Wat3	2.60	2.81	2.69	2.53

**Table 7:** Details of sodium coordination in the core of the tetramer of the Ade/2′-­dAdo complex

Ligand	Bond length (Å)
Wat1	2.60
Wat2	2.74
Wat3	2.59
Wat4	2.60
Wat5	2.48
Wat6	2.44
