# Supplementary material for: Crystallographic and SAXS studies of S-adenosyl-l-homocysteine hydrolase from Bradyrhizobium elkanii
Source: IUCrJ. 2017 Apr 10;4(Pt 3):271–82. doi: 10.1107/S2052252517002433 (PMC5414401; doi:10.1107/S2052252517002433)
Supplement: Supplementary file 1 [file m-04-00271-sup1.pdf]

IUCrJ

Volume 4 (2017)

Supporting information for article:

Crystallographic and SAXS studies on *S*-adenosyl-L-homocysteine hydrolase from *Bradyrhizobium elkanii*

Tomasz Manszewski, Kamil Szpotkowski and Mariusz Jaskolski

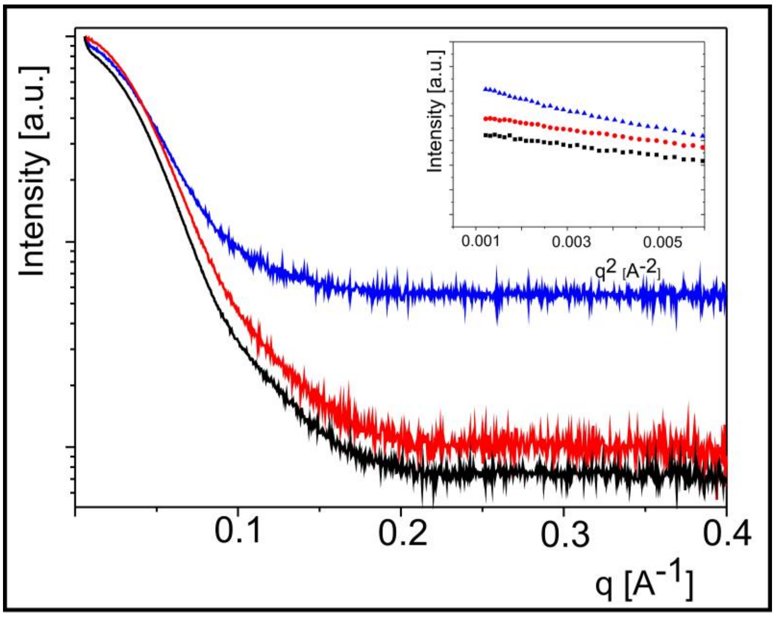

(a)

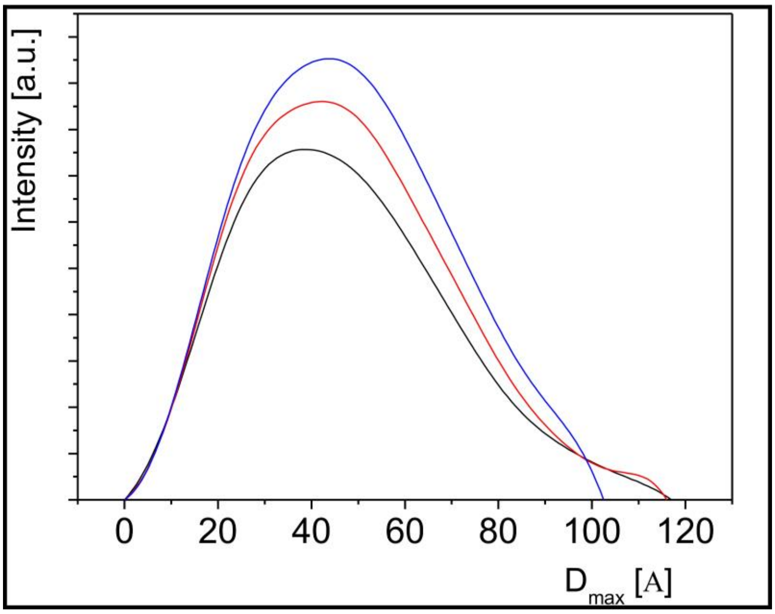

(b)

Figure S1 (a) Experimental X-ray scattering data plotted as a function of the scattering angle (black line – 1 mg ml^{-1}; red line – 2 mg ml^{-1}; blue line – 4 mg ml^{-1}); the corresponding Guinier plots are inserted in the upper right corner. (b) Pair-distance distribution functions.
